# Intraoperative ultrasound in brain tumor surgery: A review and implementation guide

**DOI:** 10.1007/s10143-022-01778-4

**Published:** 2022-03-30

**Authors:** Luke Dixon, Adrian Lim, Matthew Grech-Sollars, Dipankar Nandi, Sophie Camp

**Affiliations:** 1 Department of Imaging, Imperial College NHS Healthcare Trust, London, UK; 2 Department of Surgery and Cancer, Faculty of Medicine, Imperial College London, London, UK; 3 Department of Medical Physics, Royal Surrey NHS Foundation Trust, Guildford, UK; 4 Division of Brain Sciences, Faculty of Medicine, Imperial College London, London, UK; 5 Department of Neurosurgery, Imperial College NHS Healthcare Trust, London, UK

**Keywords:** Ultrasound, Neuronavigation, Image-guided, Oncology, Glioma

## Abstract

Accurate and reliable intraoperative neuronavigation is crucial for achieving maximal safe resection of brain tumors. Intraoperative MRI (iMRI) has received significant attention as the next step in improving navigation. However, the immense cost and logistical challenge of iMRI precludes implementation in most centers worldwide. In comparison, intraoperative ultrasound (ioUS) is an affordable tool, easily incorporated into existing theatre infrastructure, and operative workflow. Historically, ultrasound has been perceived as difficult to learn and standardize, with poor, artifact-prone image quality. However, ioUS has dramatically evolved over the last decade, with vast improvements in image quality and well-integrated navigation tools. Advanced techniques, such as contrast-enhanced ultrasound (CEUS), have also matured and moved from the research field into actual clinical use. In this review, we provide a comprehensive and pragmatic guide to ioUS. A suggested protocol to facilitate learning ioUS and improve standardization is provided, and an outline of common artifacts and methods to minimize them given. The review also includes an update of advanced techniques and how they can be incorporated into clinical practice.

**Supplementary Information:**

The online version contains supplementary material available at 10.1007/s10143-022-01778-4.

## Introduction

Maximal safe surgical resection is the core tenet of glioma neurosurgery intending to improve symptoms, quality of life, progression-free survival, and overall survival [1–3]. However, accurate tumor localization and differentiation from surrounding functional neuronal tissue remain an ongoing challenge. Preoperative stereotactic imaging (MRI/CT) is routinely used to plan surgical approaches. Although powerful tools, such systems are inherently limited as they do not offer real-time intraoperative representations of the tumor and surrounding structures. As surgery progresses, their accuracy deteriorates due to unpredictable brain shifts, distortions, and deformations [4]. Guided by non-contemporaneous inaccurate navigation, there is a risk of inadvertent damage, leading to functional deficit, or leaving residuum based on misperceived tumor margins, both impacting on prognosis [4, 5]. Consequently, there is a clear need for contemporaneous intraoperative imaging, which accurately maps the current surgical field.

Ultrasound (US) is an affordable, safe, repeatable imaging technique that can be easily integrated into surgical workflow allowing live imaging during surgery. Over the last 30 years, US has matured as a neurosurgical tool, becoming established in routine practice in many neurosurgical centers. US adoption is not, however, universal. US is difficult to standardize, highly operator-dependent, variably taught, and historically deemed a technique with poor image quality compared with CT and MRI. Given sonography’s perceived steep learning curve, attention has shifted to intraoperative MRI (iMRI) and intraoperative CT (iCT) in the last decade. These are understandably appealing tools as they are modalities with which neurosurgeons are well-versed. Despite this advantage, iCT and iMRI are still not true real-time tools and are both costly and logistically challenging. US technology has dramatically advanced in the interim, offering improved spatial and unequaled temporal resolution and making images clearer and easier to understand. Multimodal neuronavigation systems which integrate US with preoperative imaging have also greatly improved usability by assisting probe positioning in 3D space. Lastly, the development of advanced US technologies, such as contrast-enhanced US and elastography, provide additional features which are promising far better disease characterization and potential treatment guidance. This review will provide an implementation guide and update on the use of US in brain tumor surgery.

## US Physics

Diagnostic ultrasound employs a piezoelectric transducer to convert electrical signals into sound waves at above audible frequencies (between 1-20 MHz). These acoustic pressure waves are transmitted into tissue and either absorbed, scattered, or reflected, based on the wavelength and frequency of the wave and the inherent physical acoustic qualities of the tissue. The sound waves reflected as echoes are detected by the same piezoelectric transducer and converted to an electrical signal. This signal is processed into a grey-scale, 2D brightness-mode (B-mode) image, which is based on the amplitude of the echoes and the time delay between the emitted pulse and received echo.

Ultrasound propagation depends on the tissues acoustic impedance (Z), which is determined by the product of the tissues density (ρ) and the velocity of sound (c), which relates to the elasticity of the tissue (Z = ρc). Reflection of sound waves occurs at tissue interfaces where there is a change in acoustic impedance. The echogenicity of structures on US relates to the amplitude of the reflected signal, which is proportional with an interfaces acoustic gradient. For instance, choroid plexus has a high acoustic gradient with the adjacent brain and is hyperechoic, while the low density, low acoustic impedance, homogenous CSF-filled ventricles are hypoechoic. The majority of echoes in the body arise from smaller interfaces known as diffuse reflectors, which account for the characteristic speckled echotexture seen on US in different tissues. In addition to reflection, acoustic energy is also attenuated predominantly by absorption as heat and refraction. The higher the US frequency, the better the resolution but, the greater the degree of attenuation. Resultantly high-frequency probes are best for detailed imaging of superficial structures, and low-frequency, lower resolution probes are superior for visualizing deeper structures and providing a greater field of view.

## Optimizing image quality

**Fig. 1:**
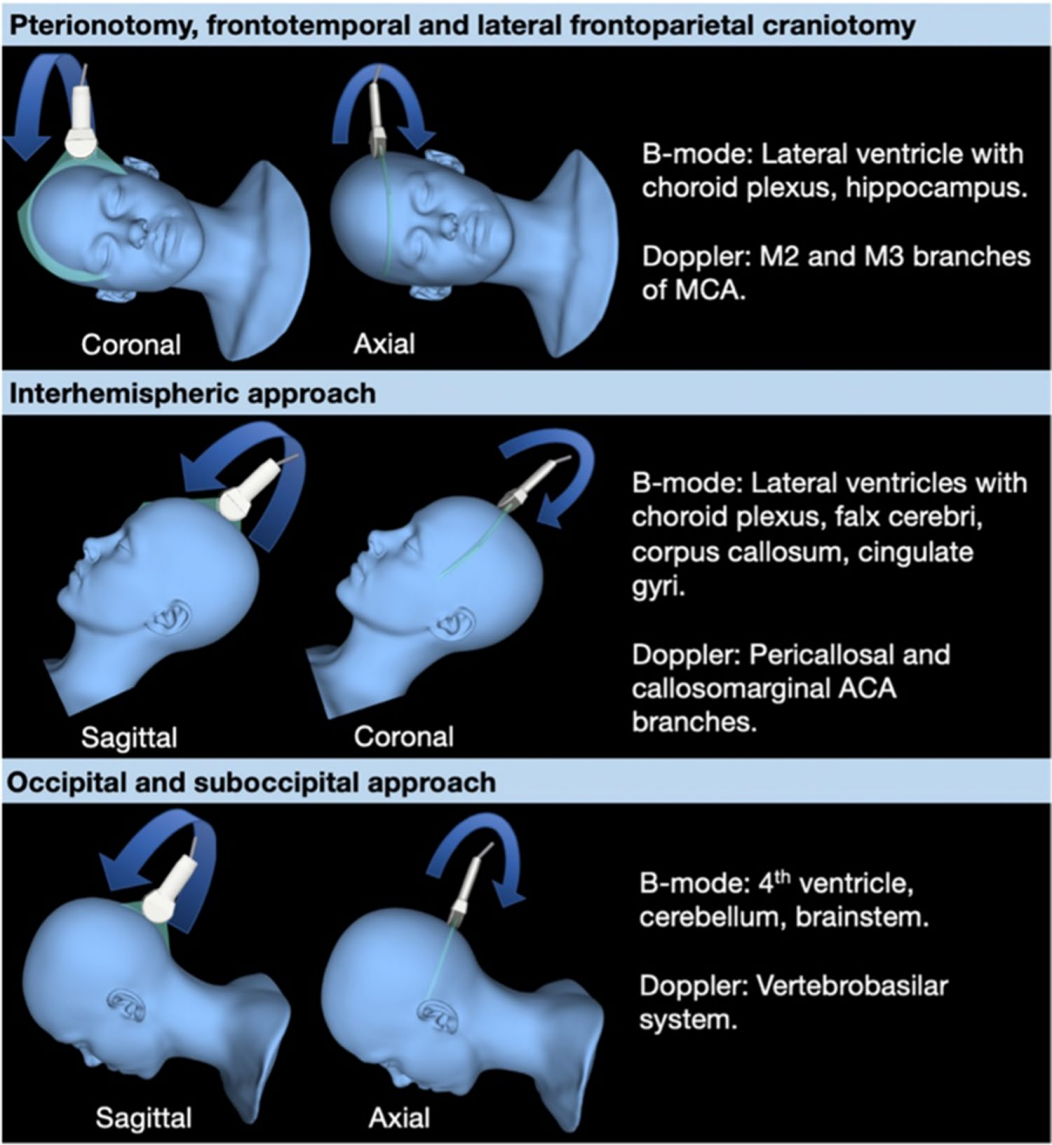
Recommended orthogonal ultrasound fans for different craniotomies with expected anatomical and vascular landmarks. Model of orthogonal ultrasound sweeps for common craniotomy sites. Probe positioned to achieve views that approximate to standard anatomical planes on CT/MRI. Patient positioned to ensure the craniotomy is as horizontal as possible to allow retention of fluid in the resection cavity for optimal ultrasound coupling

In any image-guided procedure, optimal image quality is essential for optimal accuracy. A study of 142 US-guided glioma resections found good image quality was an independent factor for more accurate estimates of resection and greater gross total resection. Conversely, poor image quality was associated with significantly worse postoperative functional outcome [6]. Image quality in US is highly variable and operator-dependent. Unlike CT/MRI, which can image the entire head in three dimensions, intraoperative ultrasound (ioUS) is restricted to the craniotomy from which there is a limited field of view. Thus uniquely, US has the potential for infinite different brain views, which are dependent on craniotomy location, probe type and probe orientation. To the uninitiated, the unfamiliar perspective and tomographic representation can be bewildering. The many different probes, settings, and potential artifacts steepen this learning curve. Formal training in neurosurgical ultrasound is also variable, and gaining experience outside the operating theatre can be difficult. With careful preparation and a consistent approach, good image quality can be achieved. To encourage a systematic approach and facilitate comparison between US and MRI, we suggest performing US sweeps in two orthogonal planes approximated to conventional anatomical planes (Fig. 1). Standardization is also needed to allow comparability and generalization between different operators and units. Extending from this, we are assessing the role of US in glioblastoma resection in the UK-based randomized controlled trial titled Functional and Ultrasound-Guided Resection of Glioblastoma (FUTURE-GB). This trial evaluates the impact of US and diffusion tensor imaging-guided resection on deterioration-free survival. To inform this trial, we have developed a suggested protocol for US-guided brain tumor resection based on our and other institutes’ experience (see supplementary material). [7].

### Probe choice

**Fig. 2:**
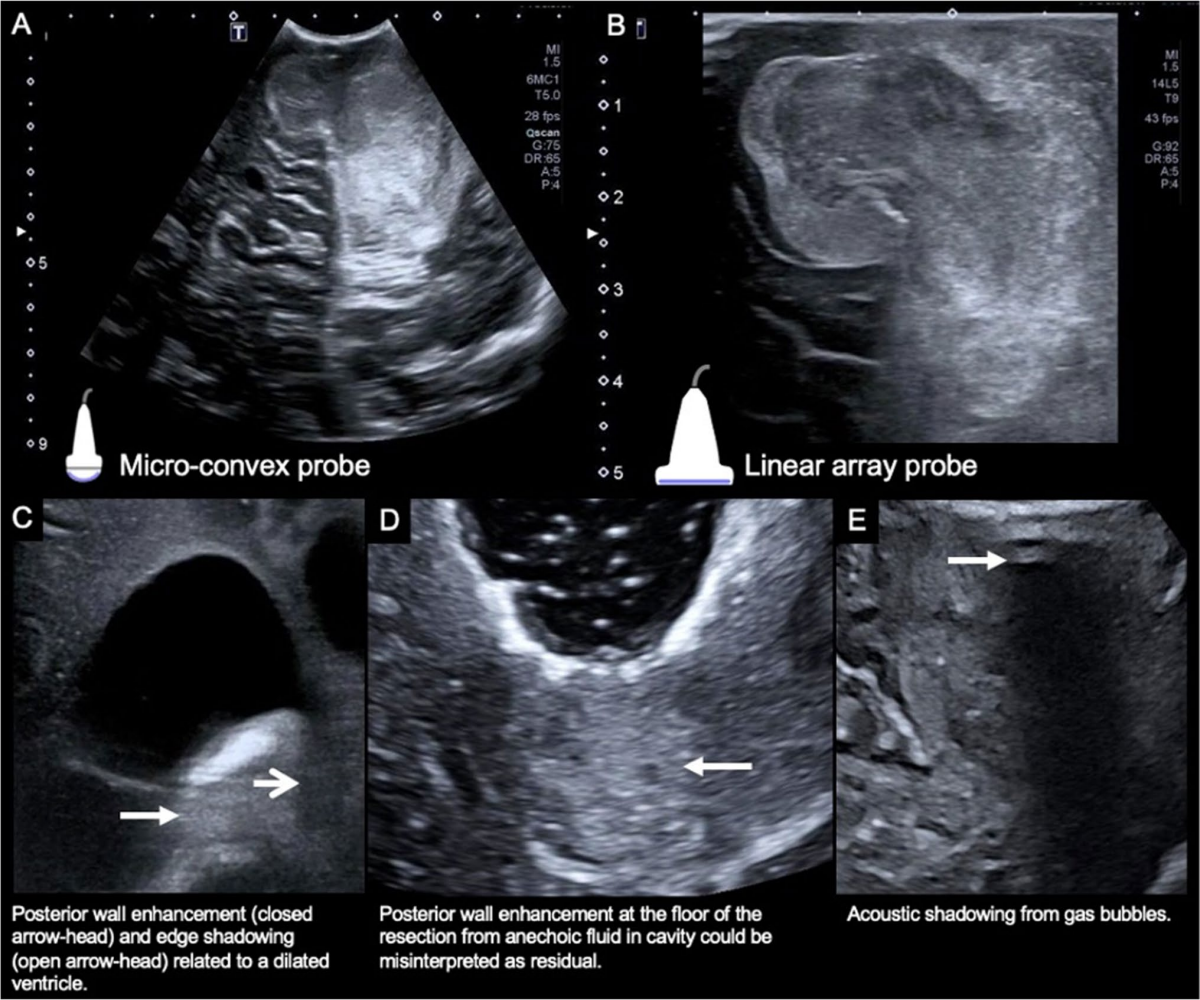
Example of ultrasound images from different probes and common ultrasound artifacts. Microconvex (**A**) and linear (**B**) US probe images of a medulloblastoma in the left cerebellum. Note the large field of view permitted by the microconvex probe (A) but the relatively poor resolution compared to the small field of view image arising from the linear probe (B). Posterior wall enhancement (closed arrowhead) and edge shadowing (open arrowhead) related to the frontal horn of the lateral ventricle (**C**). Posterior wall enhancement at the floor of a resection cavity secondary to anechoic fluid in the resection cavity could be misinterpreted as residual disease (**D**). Acoustic shadowing from gas bubbles obscures the central field of view (**E**)

There are various probes, each with different strengths and limitations (Table 1). Generally, small footprint probes are favored for intraoperative use as the craniotomy can accommodate them. There are three main types of transducer: linear, curved, and sector array. Historically, linear and curved transducers had large footprints and were limited to large craniotomies. One of the most widely used probes is a type of sector array transducer known as a phased array, a low-frequency, small foot-print probe, through which a large trapezoid field of view of the brain can be produced through a small craniotomy window. Unfortunately, phased array probes are low resolution and are particularly vulnerable to image deterioration. Recently, smaller footprint linear and curved array probes have become available, which offer better resolution. In a series comparing iMRI to a conventional phased array probe and a small footprint linear probe, sensitivity for tumor residual by linear ultrasound (79%) was almost equivalent to iMRI (83%) and far superior to the conventional phased array probe (21%) [8]. Linear probes also demonstrate significantly better residual detection than the phased array probe, with an improved extent of resection (EOR) in 75% of cases [9]. Due to the high frequency of linear probes their use is, however, limited to a depth of 4-5 cm (Fig. 2). Depending on the application, a combination of different complementary probes is thus recommended. At our institute, we employ two probes, a micro-curved and a small foot-print linear. The micro-curved transducer is an excellent compromise between size, good resolution, and large field of view. While the linear transducer is particularly useful for intracavitary scanning to assess for residual disease.

**Table 1:**
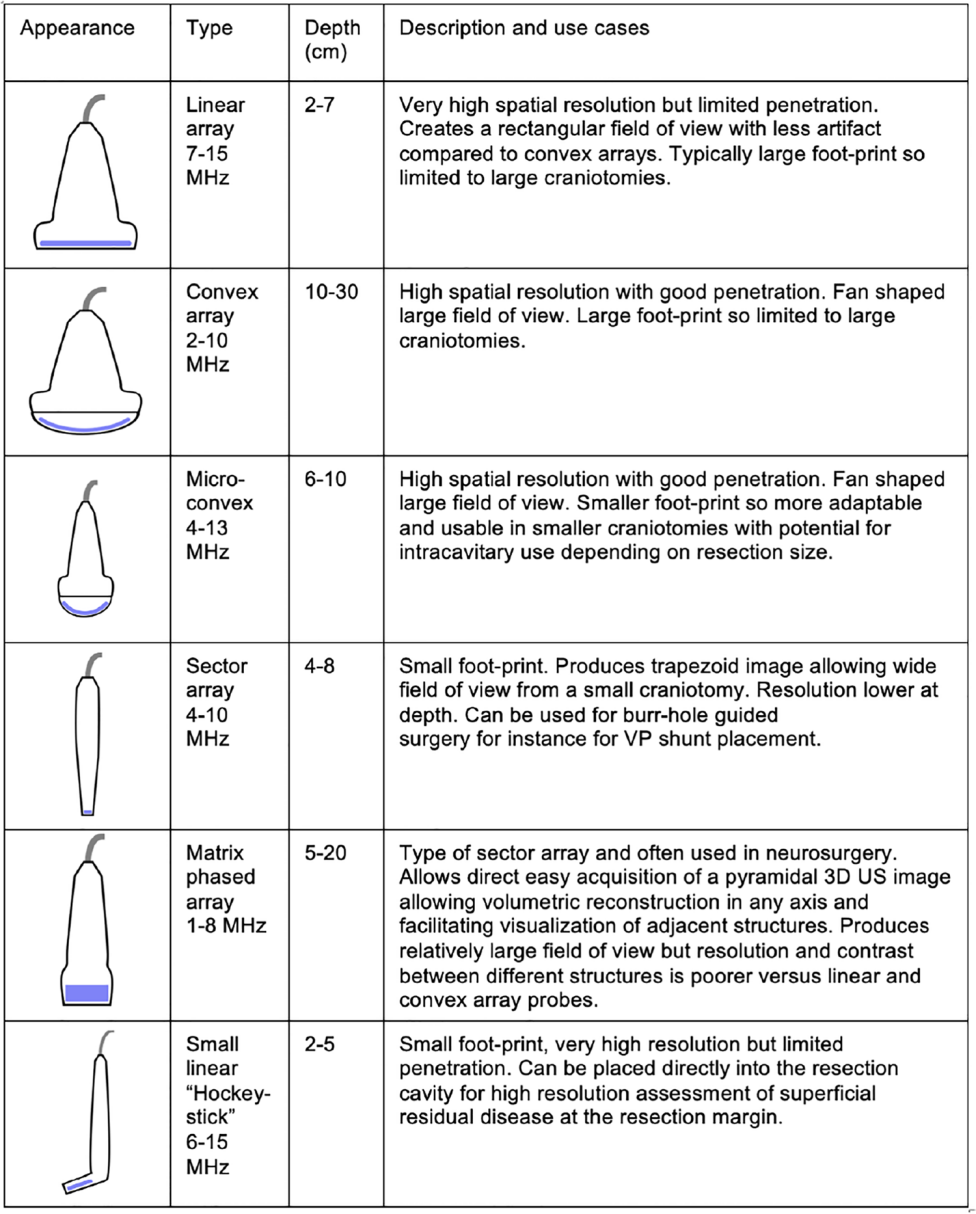
Summary table of different types of ultrasound probes and potential use cases

## Artifacts

Imaging artifacts could result in missed residual disease or, conversely damaging, over-aggressive resection of normal brain that has been misrecognized as tumor. The most frequently encountered artifacts are acoustic shadowing (AS) and posterior wall acoustic enhancement (PAE) (Fig. 2) [10]. AS occurs deep to interfaces where there is complete reflection or absorption of US, typically when there is a marked acoustic gradient or where structures strongly absorb acoustic energy, for instance, at the brain-skull interface. Gas bubbles in the surgical site or trapped within the sheathed ultrasound probe are a common source of AS. Gas bubbles can also cause ring-down artifact, which occurs when an ultrasound pulse encounters small fluid collections trapped between several gas bubbles. This trapped fluid resonates, producing a continuous signal back to the transducer, which generates an echogenic “step-ladder” like artifact shadow. Hemostatic material is particularly recognized as a cause of ring-down artifact as it can retain multiple gas locules. In a series of 15 glioma resections, ring-down artifact from gas bubbles intrinsic to Surgicel was blamed for obscuring residual tumor on US in 2 cases [11]. Edge-shadowing is another artifact that appears as a linear vertical shadow which runs parallel to the US beam at the margins of anechoic structures, such as the ventricular wall [12]. In all cases, these artifacts can usually be reduced with careful preparation (see suggested protocol in supplementary material). Furthermore, providing the operator recognizes the artifact they can usually examine the obscured region with altered probe positioning and angling.

PAE appears beneath fluid containing homogenous structures, such as cysts and fluid-filled resection cavities. Fluid attenuates US less than solid tissue, consequently deep to fluid, there are stronger sound beams that generate echoes of greater amplitude and thus greater echogenicity. Differentiating residual echogenic tumor from PAE can be difficult at the floor of a resection cavity. In a study that took samples from resection margins, there was sonographic-histological concordance at 84% of sites thought to be tumor on US, and at 88% of sites that appeared negative for tumor. The false positives were ascribed to PAE and hyperechogenic clot and contusion [13]. PAE occurs parallel to the US beam and is often linear in morphology; thus, moving the US probe and careful assessment of changes in its appearance can facilitate detection. Tangential placement of the probe on adjacent preserved cortex angulated toward the floor of the resection can also reduce PAE. Extending from this, some advocate using a separate burr hole as a dedicated US window to continuously monitor the resection in the adjacent craniotomy [14]. Intracavitary small footprint linear array and micro-convex probes are also helpful, as they can be placed directly on the resection margin, removing the artifact producing interposed fluid interface [10]. To further reduce PAE, a novel acoustic coupling fluid with an acoustic impedance similar to brain tissue has been developed and is currently being trialed with promising preliminary results. [10].

Coagulated blood, contusion, and edema also alter the appearance of the surgical field. Blood and contusion are particularly challenging as these appear echogenic, with features similar to residual disease. Intracavitary linear transducers are better at discerning residuum from other surgery-related changes [8]. The morphology of the echogenic foci is also discriminating. One study found hyperechoic foci which project irregularly into normal-appearing brain were histologically proven tumor in 89% of cases, while the homogenous, hyperechoic, even rims often observed at the resection cavity are more indeterminate, only harboring confirmed tumor in 56% of cases [15]. Advanced US techniques, such as CEUS, are also helpful in discriminating residuum and are discussed later. Finally, careful correlation with the preoperative navigation MRI and prior earlier US scans is essential, as tumor will be present on all images, while artifacts, such as PAE and surgical changes, will have developed over the surgical period. [10].

## US Navigation

Unconventional views represent a key challenge in ioUS. This is unlike navigation MRI/CT, which is easy to orientate as it provides a complete brain view. Navigated US can be carried out in either 2D or 3D and integrates with the preoperative MRI/CT so that the strengths of both systems act synergistically to overcome each other’s respective stand-alone weaknesses. [16].

### Navigated 2D-US (n2DUS) versus navigated 3D-US (n3DUS)

n2DUS fuses a live 2D-US image with the navigation MRI/CT by integrating a navigation localizer with the US probe itself. This allows navigation systems to map the probe’s position and its line of sight onto the MRI/CT [17]. In comparison to standalone US, n2DUS offers better orientation, improved delineation of tumor boundaries and more accurate detection of residuum [18, 19]. n2DUS is limited to one image plane; therefore, serial sweeps in different rotations are required to achieve multiplanar views [17]. This can be confusing when trying to colocalize a structure in 3 dimensions. Another issue is that the 2D-US probe occupies the surgical field; this can interfere with surgery by limiting access and obscuring the placement of surgical instruments [7, 17, 20]. Finally, it can be difficult to compare serial 2D-US scans as inevitably they will not be precisely coplanar.

n3DUS overcomes these issues by allowing the acquisition of 3D-US volumes, which can be retrospectively manipulated. A 3D-US volume can be reconstructed by either a manual sweep of a 2D probe which acquires around 200–300 images, or through using a matrix-phased array probe that uses beam-steering to sample points through a pyramid-shaped volume [21]. The 3D volume acquired by a 2D probe is usually higher resolution but is more subject to acquisition errors and distortion due to requiring a manual sweep. Similar to n2DUS, the acquired 3D-US volume can be directly fused with the navigation CT/MRI. Unlike n2DUS, however, once the volume has been acquired, the US probe can be put aside, and the US volume can be manipulated and used as a guide [17]. This technique compensates for the limited field of view and allows multiplanar reconstruction along conventional anatomical planes, making orientation and recognition of brain versus glioma easier [22]. Furthermore, it allows free access to the surgical field as the probe is not in-situ [20]. n3DUS also facilitates the comparison of serial US volumes, making assessing changes in the surgical field easier. In some institutions, this comparison between serial 3D-US volumes is favored and relied upon more in the late stages of resection versus comparison to the preoperative navigation MRI [17, 23]. In a series of 16 patients who underwent resection of cerebellar lesions, n3DUS showed a 71% sensitivity for detecting residual tumor [24]. In a study comparing the accuracy of n3DUS to MRI-guided navigation, the authors reported a high concordance of n3DUS with final histopathology (74 to 100% depending on tumor type). Residual detection was comparable to MRI navigation, and in low grade gliomas n3DUS was found superior to MRI [25]. US-MRI co-registration also permits fusion of preoperative DTI and fMRI data with US, allowing functional information to be overlaid with real-time anatomical data, with the aim to improve maximal safe resection. [26].

### US-MRI fusion and brain shift compensation

**Fig. 3:**
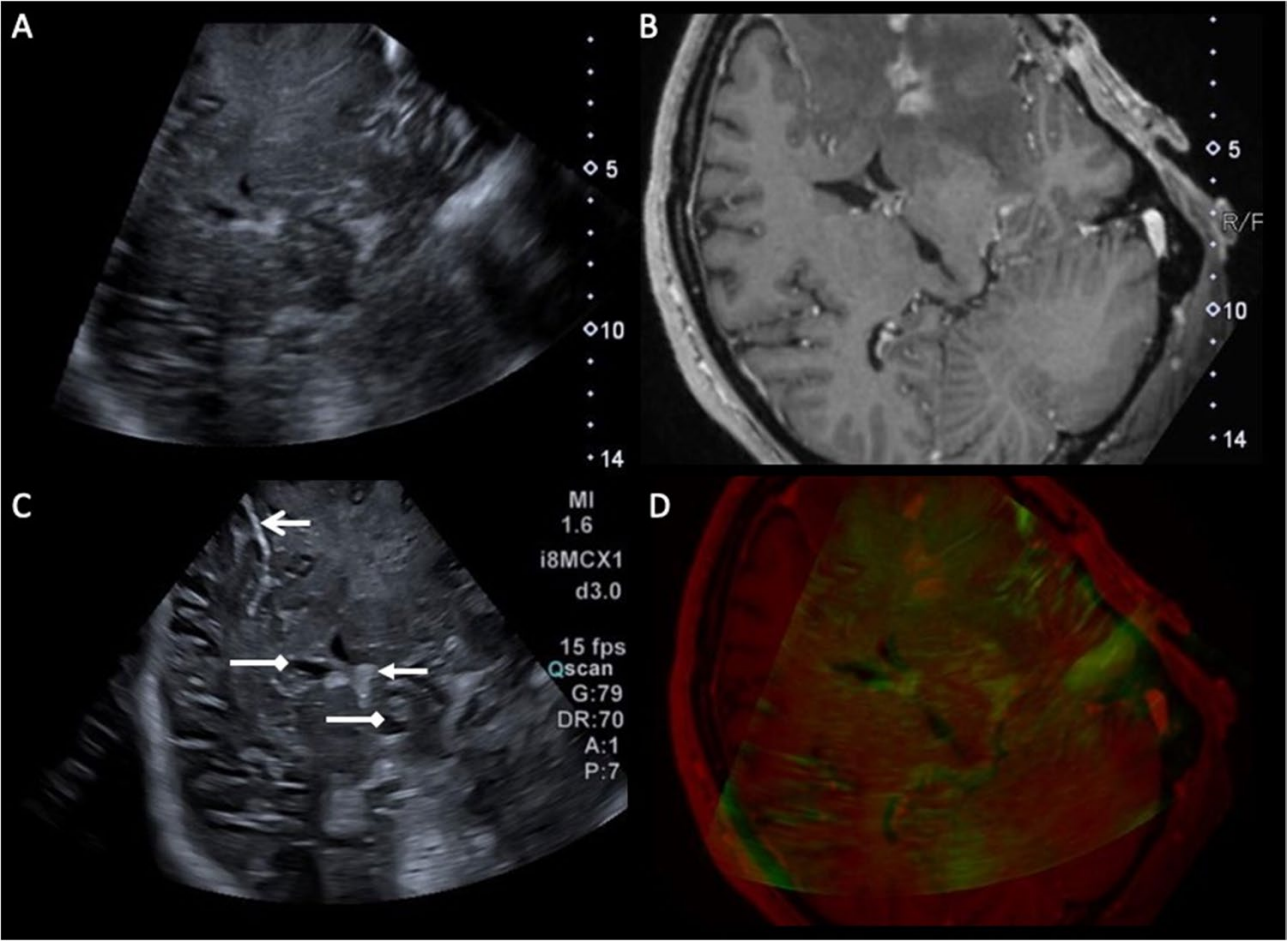
Example of navigated 3D ultrasound fused with MRI and useful anatomical landmarks. Navigated 3D ultrasound fused with MRI using rigid registration of anatomical features between a 3D ultrasound dataset (**A**) and the preoperative post contrast T1w navigation MRI (**B**). Live 2D ultrasound image (**C**) registered with the fused 3D US-MRI images (A,B) with the position of the probe tracked by an electromagnetic sensor. Overlay of the 2D ultrasound image on MRI showing quality of fusion (**D**), with the green mask representing hyperechoic structures on the US. Note the relatively poor sonographic definition of the tumour which is greatly augmented by fusion with the MRI. Useful anatomical landmarks for orientation labelled on (C): hyperechoic falx (open arrow head), lateral and 3^rd^ ventricles (top and bottom chevrons respectively) and choroid plexus in the lateral ventricle (closed arrow head)

As surgery progresses, the accuracy of US-MRI fusion deteriorates due to brain shift and increasing deformation. Brain shift alone can be significant, with studies showing a shift of up to 15 mm. [27] Several different registration techniques and algorithms have been explored to try and correct for this [7]. Rigid registration techniques are most used in practice which involves updating and co-registering common anatomical landmarks, such as the choroid plexus and falx (Fig. 3). This is best performed with the US probe in the same plane as the predominant direction of shift [7]. While this can partly account for shift in a single direction, it does not account for the highly variable and multi-directional deformation that can occur secondary to other factors like loss of CSF, re-expansion of compressed brain and changes in cerebral blood volume. Several advanced computational non-rigid registration methods have been explored to account for this inelastic deformation [28–31]. While these tools are promising, accuracy and consistency are currently variable, and it remains computationally time-consuming, limiting it to a postoperative exercise that precludes real-time correction. Considering this, several experienced institutes now advocate using the US-MRI co-registration for general orientation and the real-time US alone for assessing residual disease. [32].

## US appearance of lesions

Due to the many potential variables in US imaging, it is impossible to quantify different lesions' echogenicity objectively. Therefore, the appearance of different structures and lesions should be considered in relative degrees of echogenicity (Table 2). Simplistically, the echogenicity of intracranial tumors is dependent on cellular density. The solid components of intracranial tumors are typically moderately hyperechoic, with high-grade gliomas (HGG) and metastasis usually slightly more echogenic compared to low-grade gliomas (LGG). Generally, HGGs and metastases are also more heterogeneous in echotexture, with variable areas of necrosis (moderately hypoechoic), cysts (very hypoechoic) and hemorrhage (variable echogenicity depending on age).

**Table 2:**
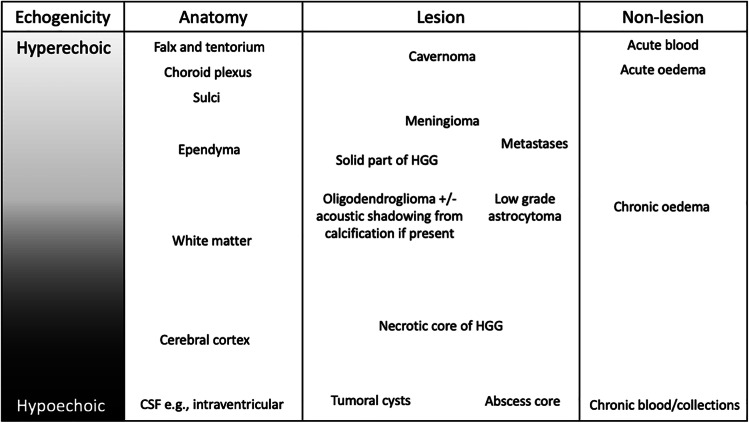
The relative echogenicity of different intracranial anatomical structures and pathologies [33]

**Fig. 4:**
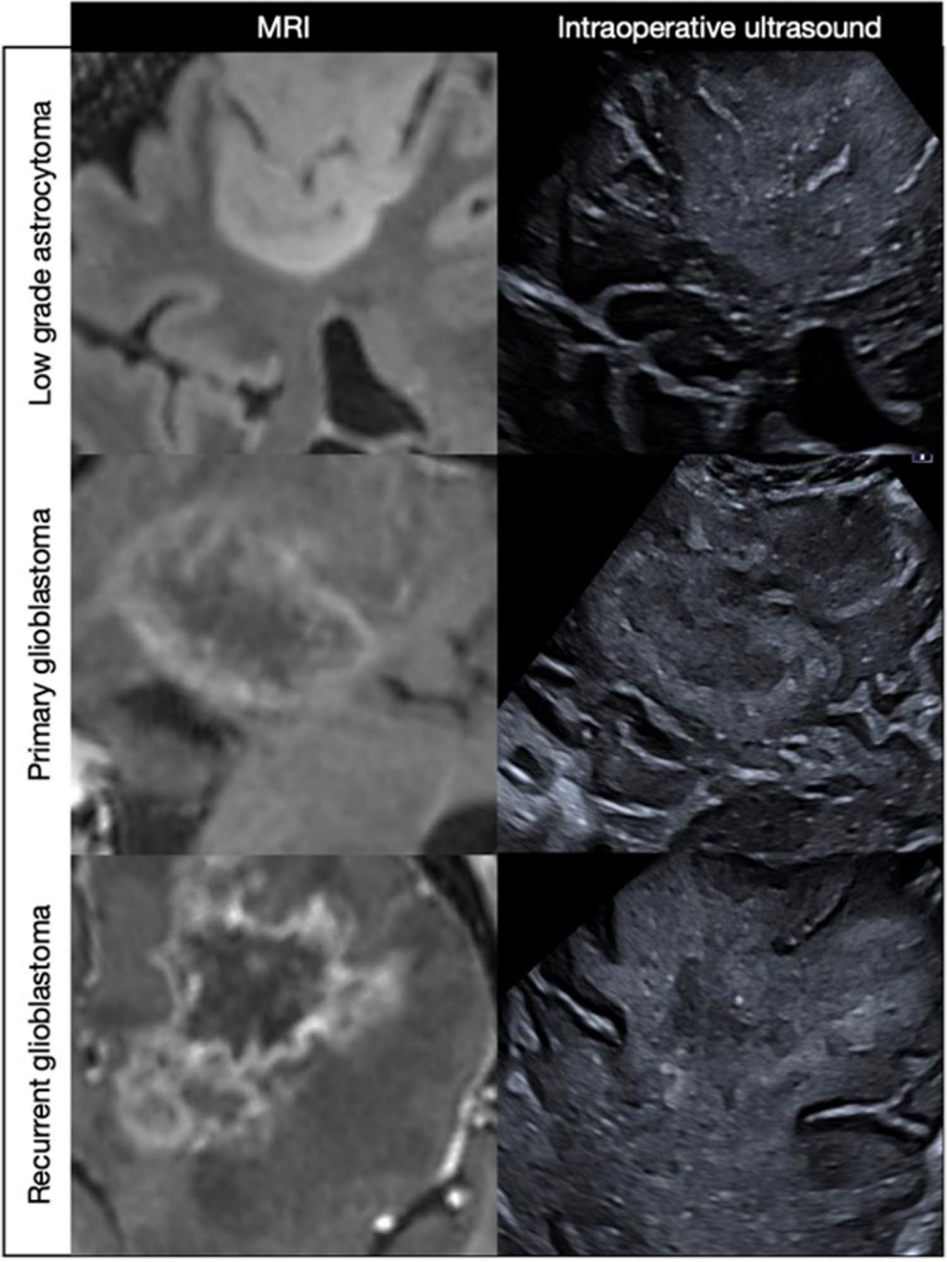
Examples of different lesions on MRI and corresponding fused ultrasound. The primary glioblastoma is the most easily visualized with clear echogenic boundaries that correlate with the enhancing rim on the preoperative post-contrast MRI. Similarly, the low-grade astrocytoma has well-defined FLAIR boundaries with corresponding matching borders on ultrasound. The recurrent glioblastoma is well defined on the post-contrast MRI but poorly visualized on ultrasound

The visibility and margins of a lesion are dependent not only on ultrasound quality but also the degree of difference in echogenicity between the lesion and surrounding brain parenchyma and the extent of infiltration. The irregular, solid, MRI enhancing components of glioblastoma and metastasis are usually well-circumscribed on ultrasound. In contrast, extensive infiltrative diffuse low-grade astrocytomas, grade III gliomas and recurrent glioblastoma after prior surgery and chemoradiotherapy are often less well visualized, with poor boundaries (Fig. 4). Similar to MRI, it can also be challenging to discriminate infiltrative tumor from surrounding edema, as both can be hyperechoic. In our experience with LGGs, if the preoperative FLAIR shows a well marginated homogenous lesion, then the US will show a matching well-defined hyperechoic lesion. While if the LGG has poorly defined margins on FLAIR with graduated abnormal signal that merges with surrounding white matter, the lesion is also likely to be poorly defined on US.

## Advanced techniques

### Doppler

**Fig. 5:**
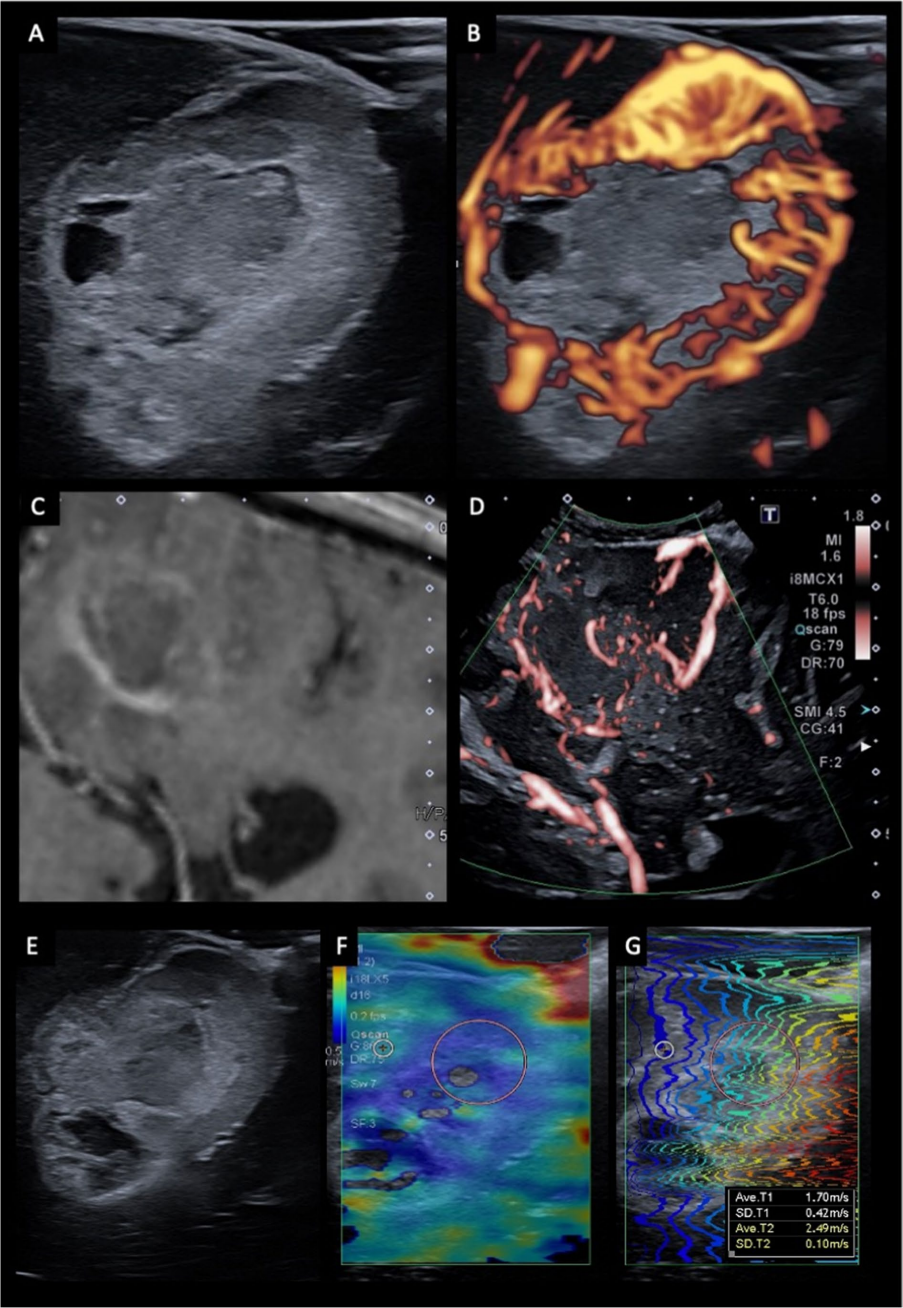
Advanced ultrasound imaging techniques. Examples of US doppler using superb microvascular imaging (SMI) (A-D) and elastography (E–G). B-mode (**A**) and doppler (**B**) imaging of a glioblastoma shows a relatively well-circumscribed, hyperechoic tumor with small hypoechoic areas in keeping with cystic necrosis (A). The tumor exhibits marked peripheral hypervascularity in keeping with neoangiogenesis (B). Post-contrast T1-weighted MRI (**C**) and doppler imaging (**D**) of a glioblastoma demonstrates the ability of US doppler imaging to resolve and identify small pial and parenchymal vessels (red) that are not appreciable on MRI. B-mode US imaging (**E**) and shear wave elastography (**F**, **G**) of a glioblastoma. The tumor demonstrates reduced stiffness compared to the surrounding brain, which is reflected by a heat map with blue reflecting low stiffness and red high stiffness

Doppler ultrasonography uses the Doppler effect to image and measure moving blood flow. The Doppler effect refers to the change in frequency that occurs when a wave reflects off an object moving toward or away from the transducer. By measuring the frequency shift generated by moving blood, the speed and direction of blood flow can be calculated. There are two main types of doppler used in oncological neurosurgery—color and power spectral doppler [33–35]. Color Doppler (CD) provides a color map of blood flow which is typically overlaid on the B-mode imaging. Conventionally, red represents blood flow toward the transducer, while blue represents blood flow away from the transducer. CD is particularly useful pre-resection before opening the dura, as it can help localize critical vessels in relation to the tumor. It provides an assessment of tumor vascularization and the location of feeding arteries and draining veins. CD is subject to aliasing artifact at depth and can become less sensitive to flow when the doppler box overlay is large [34]. Power Doppler (PD), unlike color doppler, is not affected by the angle and direction of flow, which means it is more sensitive to smaller and deeper vessels and is less subject to artifact. The trade-off with PD is that there is less information about the direction and velocity of flow. PD is, therefore, most useful when trying to detect low flow vessels and vessels deep to tumors [33, 34]. The ability of Doppler to visualize slow flow and small vessels has been recently improved with new advanced techniques, such as superb microvascular imaging (SMI), which can filter out motion artifact (Fig. 5).

### Contrast-enhanced US

**Fig. 6:**
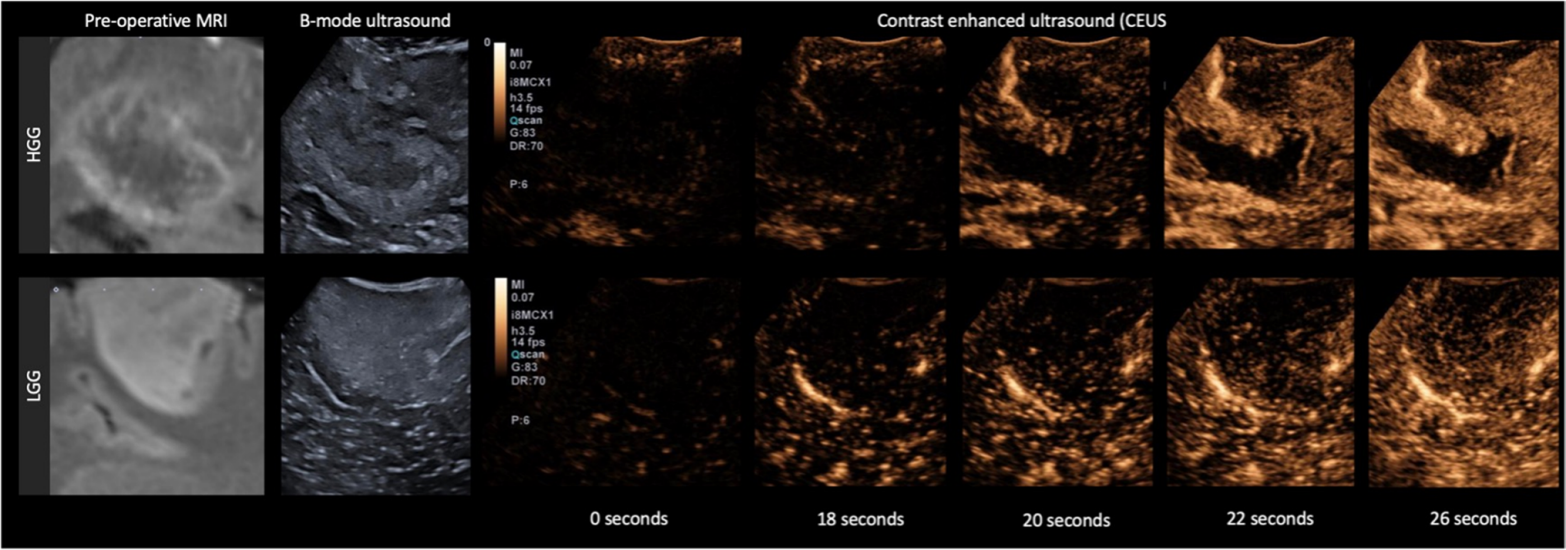
Contrast enhanced ultrasound of a high grade glioma versus a low grade glioma. Comparison between a glioblastoma (top row) and low-grade astrocytoma (bottom row). The glioblastoma demonstrates rapid arterial phase enhancement with early filling of a feeding artery at 18 s and prompt peripheral enhancement at 20 s which reaches peak at around 26 s. In comparison, the low-grade astrocytoma exhibits slow speckled enhancement, similar to the surrounding brain

The advent of contrast-enhanced US (CEUS) and its recent inclusion in the European Federation of the Societies for Ultrasound in Medicine and Biology (EFSUMB) guidelines has seen a renewed interest in the use of US in oncological neurosurgery [36]. CEUS uses intravenously injected gas-filled microbubbles, which have a high degree of echogenicity compared to surrounding soft tissues. CEUS has been used for decades in cardiology and in assessing lesions elsewhere, e.g., in the liver. Apart from very rare reports of allergic reaction, there are no known complications. Unlike gadolinium and iodinated contrast agents, microbubbles are known as “blood pool” agents as they stay intravascularly. CEUS can, therefore, both highlight tumor, help define feeding arteries and draining veins and characterize a tumor’s microvasculature and perfusion (Fig. 6). Several studies have demonstrated improved visualization and delineation of tumor boundaries versus standard B-mode [37–40]. In HGG, the contrast-enhancing regions shown on US correlate well with the preoperative gadolinium-enhanced T1-weighted images both morphologically and in volume [39]. Furthermore, both the pattern of contrast enhancement and quantitative analysis of perfusion can help differentiate glioma grade [41]. LGGs exhibit minimal to mildly greater enhancement relative to normal brain, with indistinct margins and organized perfusion similar to adjacent parenchyma. HGGs demonstrate avid contrast enhancement with rapid arterial perfusion and venous drainage with disorganized vascularity [37, 42]. There is limited evidence that CEUS may facilitate discriminating tumor from radiation necrosis, with the latter noted to be non-enhancing on CEUS [43]. CEUS is recommended at the start of surgery before opening the dura to define the boundaries of the enhancing tumor and characterize the enhancement characteristics of the tumor [44]. Enhancement characterization serves two purposes: 1) it gives a baseline impression of how enhancing the tumor is, which determines whether CEUS will be sensitive to residual tumor on repeat scanning (i.e., CEUS will be less sensitive in poorly enhancing tumors) and 2) it allows mapping of high perfusion regions that warrant removal or sampling, as they are likely to be representative of the highest grade components. If the tumor exhibits enhancement, a second CEUS scan at the end of resection is recommended to check for residual enhancing disease. CEUS has been proven helpful in detecting residual following HGG resection, with greater sensitivity versus conventional B-mode [41, 45, 46]. One series found CEUS had a synergistic role with 5-ALA, noting the best EOR with both techniques combined rather than either technique in isolation. The authors found CEUS improved detection of 5-ALA occult residual obscured by blood products, overlapping tissue or incomplete illumination [47]. This observation of improved EOR with CEUS is mirrored by several other series [46, 48].CEUS has a few drawbacks, firstly implementation requires training and can be logistically challenging at the start, secondly, slow flow vessels and devascularized tumor can be concealed and finally, the sensitivity of CEUS in previously treated tumor may be reduced, with one study noticing a false positive rate in two-thirds of cases treated with prior radiotherapy. [49].

### Elastography

Manual palpation remains a valuable tool to help differentiate tumor from surrounding parenchyma, relying on the inherent differences in compressibility and stiffness. Elastography is an imaging-based extension of this concept, which assesses the relationship between stress (the mechanical force applied) and strain (the proportional deformation) according to Young’s E modulus [33]. There is a range of elastography techniques, with compression or strain elastography (SE) and shear-wave elastography (SWE) the most used in clinical practice. SE is a qualitative technique that involves acquiring US before and after applying a deformation force. This external force can either be generated manually using fluctuating gentle pressure with the US probe or by holding the probe in a fixed position against the parenchyma allowing for the deformation to be generated by normal cerebral pulsation [50]. SE is, however, highly operator dependent and there are concerns regarding reproducibility. In comparison, SWE is a quantitative technique that uses focused acoustic radiation to generate transverse shear waves that cause tissue displacement (Fig. 5). These can be mapped to calculate shear wave velocities and measures of stiffness (in kPa) [51]. Presently, elastography remains in the research realm, with its role in neurosurgery uncertain. Studies have reported improved definition of tumor margins versus B-mode for both SE and SWE, and there have been case reports of SWE being used to identify MRI occult areas of focal cortical dysplasia [50, 52]. Elastography may also aid discriminating disease grade, as LGGs are generally observed to be homogenously stiffer than adjacent parenchyma, while HGGs are usually softer than neighboring brain [50]. Despite these advances, translation of elastography into clinical practice remains hampered by operator dependence, challenging interpretation and uncertain correlation with histology. [53].

## Conclusion

To achieve maximal safe resection, there is an exceptionally fine line between tumor removal and preserving neurological function. Presently, there is no single tool that bridges this gap. There is, however, an increasing body of evidence that US can be a useful addition to the intraoperative neurosurgical tools, as it provides real-time mapping of the surgical field. Nevertheless, its widespread adoption has been limited by a perceived steep learning curve, unfamiliar artifacts, and variable image production and interpretation. With a systematic approach, appropriate training, and the implementation of the latest US technologies, these difficulties can be greatly ameliorated. Moving forward, there is a need for larger, more robust studies to assess the impact of US on brain tumor resection and for greater development of US technologies that are specifically targeted to neurosurgery.

## Supplementary Information

Below is the link to the electronic supplementary material.

Supplementary file1 (DOCX 26 KB)
