# Supplementary material for: Intraoperative ultrasound in brain tumor surgery: A review and implementation guide
Source: Neurosurg Rev. 2022 Mar 30;45(4):2503–15. doi: 10.1007/s10143-022-01778-4 (PMC9349149; doi:10.1007/s10143-022-01778-4)
Supplement: Supplementary file 1 — Supplementary file1 (DOCX 26 KB) [file 10143_2022_1778_MOESM1_ESM.docx]

**Supplementary Material**

*Proposed IOUS protocol*

1. Patient set-up:
   1. Aim to position the patient so that the craniotomy is directed upwards and is horizontal (parallel to the floor). This allows saline retention to ensure good contact with the US probe and to reduce the presence of artifact-producing gas.^21,53^
   2. Ensure that there is sufficient space for the US machine to be positioned on one side of the patient.
2. Scanner set-up:
   1. Select appropriate probes (see Fig. 1): Generally, at least two small footprint probes are recommended - one low-frequency curved or phased array transducer for moderate resolution but large field of view imaging, and one high-frequency linear transducer for high resolution, detailed superficial imaging.
   2. Probe preparation: sterilize probes before surgery or use disposable, sterile probe sheaths. At our institute, probe sheaths are favored, as this allows fast turnaround between serial cases without the need for time-consuming sterilization or the cost of duplicate probes. Complete coverage of the probe head with sterile ultrasound gel followed by tight application of the sheath is recommended to prevent gas bubbles from being trapped in the sheath.^21,49^ A discussion of different US probe sterilization techniques is given elsewhere.^44^
   3. Control panel: for operation of the US machine settings, the panel can be covered with a sterile transparent drape to allow direct control by the primary surgeon. Alternatively, an assistant can adjust the settings.
   4. Fusion with neuronavigation: Depending on the neuronavigation fusion technique, registration of the US probe with the preoperative navigation imaging is possible before craniotomy.
3. Craniotomy:
   1. Using the preoperative cross-sectional (CT/MRI) neuronavigation imaging to plan the craniotomy site. As previously noted, aim to have the craniotomy horizontal (parallel to the floor) and orientated upwards.
   2. Ensure the craniotomy is sufficient in size to allow visualization of the entire lesion and to accommodate the US probe. Modern small footprint probes are easily accommodated and should not themselves usually necessitate an increase in the craniotomy extent.
   3. To better retain saline at the surgical site, some surgeons infiltrate local anesthetic in the scalp around the margins of the craniotomy. This creates a soft tissue lip which acts as a dam for saline. Similarly, one group reports the novel use of bone wax to make custom dams around non-horizontal craniotomies.^20^
4. First scan(s):
   1. Before opening the dura, an initial planning ultrasound scan should be performed as brain shift, and distortion will be minimal. Rarely there may be dural calcification which can obscure the field of view (see “Artefacts”) if present, then the following initial scans can, however, be performed after opening the dura. During the scan, fill the craniotomy site with sterile saline to allow effective coupling of the ultrasound with the dura to remove any obscuring gas. Ensure the ultrasound probe is contacting the dura/brain fully but avoid overt pressure as this will cause further deformation of anatomy and potential damage.
   2. There are two main ways to manipulate the probe in ioUS:
      1. Sweep - translation of the probe across the structure being imaged.^4^ This is generally very limited when imaging through a craniotomy.
      2. Fan – tilting of the probe at a fixed point to change the angle of insonation.^4^ In neurosurgery, this is the primary probe manipulation technique.
   3. At each scanning, interval perform two orthogonal fans approximated to conventional anatomical planes (axial, coronal, sagittal).^2^ This will present the anatomy in a familiar representation and improve consistency on repeat scanning. It will also help reorientation when retrospectively reviewing saved images which may be necessary to differentiate artifact or residuum (see “Artefacts”).^2^ A summary of recommended orthogonal planes in different craniotomies is given in Figure 3.
   4. Start with two large orthogonal field of view ultrasound sweeps using the lower frequency probe. This will give an overall picture of the surgical field, visualizing the tumor and its relation to the surrounding anatomy.^55^ Subsequently, reduce the depth to make the lesion fill the majority of the ultrasound and take further smaller field of view sweeps in the same orthogonal planes. During these scans:
      1. Orientate the ultrasound view and review it relative to preoperative navigation MRI by checking for reliable anatomical landmarks, such as the falx and tentorium cerebelli (linear hyperechoic structures), ventricles (anechoic), choroid plexus (hyperechoic inside the ventricles), and major intracranial vessels (e.g. sagittal venous sinus and middle cerebral artery).
      2. If not already fused, register the ultrasound with the preoperative navigation imaging and assess the quality of the fusion by comparing anatomical and lesion landmarks. Correct for brain shift if possible (see “Ultrasound Navigation”).
      3. Assess lesion visibility and consider whether craniotomy extension is needed.
      4. Adjust ultrasound settings to optimize image quality. The main parameters that may need adjusting are depth, frequency, gain, and focus.
      5. If supported, acquire 3D ultrasounds for retrospective manipulation.
      6. Plan the surgical corridor to the lesion – mapping the site of cortectomy and assessing the feasibility of a trans-sulcal approach.
   5. After initial B-mode scans, use doppler to identify nearby vessels (arteries/veins), which may alter the surgical approach.
5. Serial interval scans:
   1. Once the dura has been opened and the resection has commenced, regular repeat ultrasound scans are recommended to assess the residual disease and look for any developing complications or artifacts which may impact interpretation and resection (see “Artefacts”). Before repeat scans, wash out the cavity and refill it with clean saline to remove obscuring debris and clot.
6. Post-resection scan(s):
   1. Repeat scans following the same method as steps 4 c-e. This allows easy comparison to the pre-resection ultrasound. Assess for resection and inadvertent surgical damage.
   2. Then use the high frequency, small footprint, linear probe in the cavity. This provides greater detail and is less subject to artifact.^10,11^ Directly sweep the probe along the resection walls systematically; for instance, imagine the cavity as a clockface and undertake serial sweeps from opposing numbers, e.g., 12 to 6 o’clock, 1 to 7 o’clock, etc.
   3. If residual is identified, which is amenable to resection (without risk of significant deficit), perform further resection and repeat step 6. To colocalize residual between the ultrasound image and the physical surgical field, a metallic probe, blunt needle, or even a tiny piece of hemostatic material can be placed under US guidance on the surface of the residual as a way of labelling before direct resection.

**References**

1. Almeida JP, Chaichana KL, Rincon-Torroella J, Quinones-Hinojosa A. The Value of Extent of Resection of Glioblastomas: Clinical Evidence and Current Approach. *Current Neurology and Neuroscience Reports*. Published online 2015. doi:10.1007/s11910-014-0517-x

2. Altieri R, Melcarne A, Di Perna G, et al. Intra-Operative Ultrasound: Tips and Tricks for Making the Most in Neurosurgery. *Surgical technology international*. Published online 2018.

3. Arlt F, Chalopin C, Müns A, Meixensberger J, Lindner D. Intraoperative 3D contrast-enhanced ultrasound (CEUS): a prospective study of 50 patients with brain tumours. *Acta Neurochirurgica*. Published online 2016. doi:10.1007/s00701-016-2738-z

4. Bahner DP, Blickendorf JM, Bockbrader M, et al. Language of Transducer Manipulation: Codifying Terms for Effective Teaching. *Journal of Ultrasound in Medicine*. Published online 2016. doi:10.7863/ultra.15.02036

5. Bal J, Camp SJ, Nandi D. The use of ultrasound in intracranial tumor surgery. *Acta Neurochirurgica*. Published online 2016. doi:10.1007/s00701-016-2803-7

6. del Bene M, Perin A, Casali C, et al. Advanced Ultrasound Imaging in Glioma Surgery: Beyond Gray-Scale B-mode. *Frontiers in Oncology*. 2018;8. doi:10.3389/fonc.2018.00576

7. Chacko AG, Kumar NKS, Chacko G, Athyal R, Rajshekhar V, Unsgaard G. Intraoperative ultrasound in determining the extent of resection of parenchymal brain tumours - A comparative study with computed tomography and histopathology. *Acta Neurochirurgica*. Published online 2003. doi:10.1007/s00701-003-0009-2

8. Chan HW, Pressler R, Uff C, et al. A novel technique of detecting MRI-negative lesion in focal symptomatic epilepsy: Intraoperative ShearWave Elastography. *Epilepsia*. 2014;55(4). doi:10.1111/epi.12562

9. Cheng LG, He W, Zhang HX, et al. Intraoperative Contrast Enhanced Ultrasound Evaluates the Grade of Glioma. *BioMed Research International*. 2016;2016. doi:10.1155/2016/2643862

10. Coburger J, König RW, Scheuerle A, et al. Navigated high frequency ultrasound: Description of technique and clinical comparison with conventional intracranial ultrasound. *World Neurosurgery*. Published online 2014. doi:10.1016/j.wneu.2014.05.025

11. Coburger J, Scheuerle A, Thal DR, et al. Linear array ultrasound in low-grade glioma surgery: histology-based assessment of accuracy in comparison to conventional intraoperative ultrasound and intraoperative MRI. *Acta Neurochirurgica*. Published online 2015. doi:10.1007/s00701-014-2314-3

12. D’Agostino E, Maes F, Vandermeulen D, Suetens P. A viscous fluid model for multimodal non-rigid image registration using mutual information. *Medical Image Analysis*. 2003;7(4). doi:10.1016/S1361-8415(03)00039-2

13. Duffau H. Long-term outcomes after supratotal resection of diffuse low-grade gliomas: a consecutive series with 11-year follow-up. *Acta Neurochirurgica*. Published online 2016. doi:10.1007/s00701-015-2621-3

14. Erdoan N, Tucer B, Mavl E, Menkü A, Kurtsoy A. Ultrasound guidance in intracranial tumor resection: Correlation with postoperative magnetic resonance findings. *Acta Radiologica*. Published online 2005. doi:10.1080/02841850500223208

15. Ferrant M, Nabavi A, Macq B, Jolesz FA, Kikinis R, Warfield SK. Registration of 3-d intraoperative MR images of the brain using a finite-element biomechanical model. *IEEE Transactions on Medical Imaging*. 2001;20(12). doi:10.1109/42.974933

16. Gerard IJ, Kersten-Oertel M, Petrecca K, Sirhan D, Hall JA, Collins DL. Brain shift in neuronavigation of brain tumors: A review. *Medical Image Analysis*. Published online 2017. doi:10.1016/j.media.2016.08.007

17. Halliwell M. Diagnostic Ultrasound: Physics and Equipment , 2nd edition . *Ultrasound*. Published online 2010. doi:10.1258/ult.2010.100018

18. Hervey-Jumper SL, Berger MS. Maximizing safe resection of low- and high-grade glioma. *Journal of Neuro-Oncology*. Published online 2016. doi:10.1007/s11060-016-2110-4

19. Hu X, Xu R, Ding H, et al. The total resection rate of glioma can be improved by the application of US-MRI fusion combined with contrast-enhanced ultrasound. *Clinical Neurology and Neurosurgery*. 2021;208. doi:10.1016/j.clineuro.2021.106892

20. *Intraoperative Ultrasound (IOUS) in Neurosurgery*.; 2016. doi:10.1007/978-3-319-25268-1

21. Ivanov M, Wilkins S, Poeata I, Brodbelt A. Intraoperative ultrasound in neurosurgery - A practical guide. *British Journal of Neurosurgery*. Published online 2010. doi:10.3109/02688697.2010.495165

22. Lindseth F, Kaspersen JH, Ommedal S, et al. Multimodal image fusion in ultrasound-based neuronavigation: Improving overview and interpretation by integrating preoperative MRI with intraoperative 3D ultrasound. *Computer Aided Surgery*. Published online 2003. doi:10.3109/10929080309146040

23. Mattei L, Prada F, Marchetti M, Gaviani P, DiMeco F. Differentiating brain radionecrosis from tumour recurrence: a role for contrast-enhanced ultrasound? *Acta Neurochirurgica*. 2017;159(12). doi:10.1007/s00701-017-3306-x

24. Mercier L, Araujo D, Haegelen C, del Maestro RF, Petrecca K, Collins DL. Registering Pre- and Postresection 3-Dimensional Ultrasound for Improved Visualization of Residual Brain Tumor. *Ultrasound in Medicine & Biology*. 2013;39(1). doi:10.1016/j.ultrasmedbio.2012.08.004

25. Miller D, Heinze S, Tirakotai W, et al. Is the image guidance of ultrasonography beneficial for neurosurgical routine? *Surgical Neurology*. Published online 2007. doi:10.1016/j.surneu.2006.07.021

26. Moiyadi A V., Shetty P. Direct navigated 3D ultrasound for resection of brain tumors: A useful tool for intraoperative image guidance. *Neurosurgical Focus*. Published online 2016. doi:10.3171/2015.12.FOCUS15529

27. Nimsky C, Ganslandt O, Cerny S, Hastreiter P, Greiner G, Fahlbusch R. Quantification of, Visualization of, and Compensation for Brain Shift Using Intraoperative Magnetic Resonance Imaging. *Neurosurgery*. 2000;47(5). doi:10.1097/00006123-200011000-00008

28. della Pepa GM, Ius T, la Rocca G, et al. 5-Aminolevulinic Acid and Contrast-Enhanced Ultrasound: The Combination of the Two Techniques to Optimize the Extent of Resection in Glioblastoma Surgery. *Neurosurgery*. 2020;86(6). doi:10.1093/neuros/nyaa037

29. della Pepa GM, Sabatino G, la Rocca G. “Enhancing Vision” in High Grade Glioma Surgery: A Feasible Integrated 5-ALA + CEUS Protocol to Improve Radicality. *World Neurosurgery*. 2019;129. doi:10.1016/j.wneu.2019.06.127

30. Pino MA, Imperato A, Musca I, et al. New hope in brain glioma surgery: The role of intraoperative ultrasound. A review. *Brain Sciences*. Published online 2018. doi:10.3390/brainsci8110202

31. Prada F, Bene M del, Fornaro R, et al. Identification of residual tumor with intraoperative contrast-enhanced ultrasound during glioblastoma resection. *Neurosurgical Focus*. 2016;40(3). doi:10.3171/2015.11.FOCUS15573

32. Prada F, del Bene M, Mattei L, et al. Preoperative Magnetic Resonance and Intraoperative Ultrasound Fusion Imaging for Real-Time Neuronavigation in Brain Tumor Surgery. *Ultraschall in der Medizin - European Journal of Ultrasound*. 2014;36(02). doi:10.1055/s-0034-1385347

33. Prada F, del Bene M, Moiraghi A, et al. From Grey Scale B-Mode to Elastosonography: Multimodal Ultrasound Imaging in Meningioma Surgery—Pictorial Essay and Literature Review. *BioMed Research International*. 2015;2015. doi:10.1155/2015/925729

34. Prada F, del Bene M, Rampini A, et al. Intraoperative Strain Elastosonography in Brain Tumor Surgery. *Operative Neurosurgery*. 2019;17(2). doi:10.1093/ons/opy323

35. Prada F, Mattei L, del Bene M, et al. Intraoperative cerebral glioma characterization with contrast enhanced ultrasound. *BioMed Research International*. Published online 2014. doi:10.1155/2014/484261

36. Prada F, Perin A, Martegani A, et al. Intraoperative Contrast-Enhanced Ultrasound for Brain Tumor Surgery. *Neurosurgery*. 2014;74(5). doi:10.1227/NEU.0000000000000301

37. Prada F, Vetrano IG, Gennari AG, et al. How to Perform Intra-Operative Contrast-Enhanced Ultrasound of the Brain—A WFUMB Position Paper. *Ultrasound in Medicine & Biology*. 2021;47(8):2006-2016. doi:10.1016/j.ultrasmedbio.2021.04.016

38. Prada F, Vitale V, del Bene M, et al. Contrast-enhanced MR Imaging versus Contrast-enhanced US: A Comparison in Glioblastoma Surgery by Using Intraoperative Fusion Imaging. *Radiology*. 2017;285(1). doi:10.1148/radiol.2017161206

39. de Quintana-Schmidt C, Salgado-Lopez L, Aibar-Duran JA, et al. Neuronavigated Ultrasound in Neuro-Oncology: A True Real-Time Intraoperative Image. *World Neurosurgery*. 2022;157:e316-e326. doi:10.1016/j.wneu.2021.10.082

40. Rasmussen IA, Lindseth F, Rygh OM, et al. Functional neuronavigation combined with intra-operative 3D ultrasound: Initial experiences during surgical resections close to eloquent brain areas and future directions in automatic brain shift compensation of preoperative data. *Acta Neurochirurgica*. 2007;149(4). doi:10.1007/s00701-006-1110-0

41. Reinertsen I, Lindseth F, Askeland C, Iversen DH, Unsgård G. Intra-operative correction of brain-shift. *Acta Neurochirurgica*. 2014;156(7). doi:10.1007/s00701-014-2052-6

42. Renovanz M, Hickmann AK, Henkel C, Nadji-Ohl M, Hopf NJ. Navigated versus non-navigated intraoperative ultrasound: Is there any impact on the extent of resection of high-grade gliomas? A retrospective clinical analysis. *Journal of Neurological Surgery, Part A: Central European Neurosurgery*. Published online 2014. doi:10.1055/s-0033-1356486

43. Rohde V, Coenen VA. Intraoperative 3-dimensional ultrasound for resection control during brain tumour removal: preliminary results of a prospective randomized study. *Acta neurochirurgica Supplement*. Published online 2011. doi:10.1007/978-3-211-99651-5_29

44. Sastry R, Bi WL, Pieper S, et al. Applications of Ultrasound in the Resection of Brain Tumors. *Journal of Neuroimaging*. Published online 2017. doi:10.1111/jon.12382

45. Selbekk T, Brekken R, Indergaard M, Solheim O, Unsgård G. Comparison of contrast in brightness mode and strain ultrasonography of glial brain tumours. *BMC Medical Imaging*. 2012;12(1). doi:10.1186/1471-2342-12-11

46. Selbekk T, Jakola AS, Solheim O, et al. Ultrasound imaging in neurosurgery: Approaches to minimize surgically induced image artefacts for improved resection control. *Acta Neurochirurgica*. Published online 2013. doi:10.1007/s00701-013-1647-7

47. Sidhu P, Cantisani V, Dietrich C, et al. The EFSUMB Guidelines and Recommendations for the Clinical Practice of Contrast-Enhanced Ultrasound (CEUS) in Non-Hepatic Applications: Update 2017 (Long Version). *Ultraschall in der Medizin - European Journal of Ultrasound*. 2018;39(02). doi:10.1055/a-0586-1107

48. Solheim O, Selbekk T, Jakola AS, Unsgård G. Ultrasound-guided operations in unselected high-grade gliomas-overall results, impact of image quality and patient selection. *Acta Neurochirurgica*. Published online 2010. doi:10.1007/s00701-010-0731-5

49. Sosna J, Barth MM, Kruskal JB, Kane RA. Intraoperative sonography for neurosurgery. *Journal of Ultrasound in Medicine*. Published online 2005. doi:10.7863/jum.2005.24.12.1671

50. Steel R, Poepping TL, Thompson RS, MacAskill C. Origins of the edge shadowing artefact in medical ultrasound imaging. *Ultrasound in Medicine and Biology*. Published online 2004. doi:10.1016/j.ultrasmedbio.2004.07.014

51. Taljanovic MS, Gimber LH, Becker GW, et al. Shear-Wave Elastography: Basic Physics and Musculoskeletal Applications. *RadioGraphics*. 2017;37(3). doi:10.1148/rg.2017160116

52. Tao A yu, Chen X, Zhang L yun, et al. Application of Intraoperative Contrast-Enhanced Ultrasound in the Resection of Brain Tumors. *Current Medical Science*. 2022;42(1):169-176. doi:10.1007/s11596-022-2538-z

53. Unsgaard G, Gronningsaeter A, Ommedal S, et al. Brain operations guided by real-time two-dimensional ultrasound: New possibilities as a result of improved image quality. *Neurosurgery*. Published online 2002. doi:10.1097/00006123-200208000-00019

54. Unsgaard G, Kleven A, Ommedal S, Gronningsaeter A. An Ultrasound-Based Neuronavigation System, A Good Solution To The Brain-Shift Problem. *Neurosurgery*. Published online 1999. doi:10.1097/00006123-199909000-00188

55. Unsgaard G, Rygh OM, Selbekk T, et al. Intra-operative 3D ultrasound in neurosurgery. *Acta Neurochirurgica*. Published online 2006. doi:10.1007/s00701-005-0688-y

56. Woydt M, Krone A, Becker G, Schmidt K, Roggendorf W, Roosen K. Correlation of Intra-Operative Ultrasound with Histopathologic Findings after Tumour Resection in Supratentorial Gliomas: A Method to Improve Gross Total Tumour Resection. *Acta Neurochirurgica*. Published online 1996. doi:10.1007/BF01411117

57. Yeole U, Singh V, Mishra A, Shaikh S, Shetty P, Moiyadi A. Navigated intraoperative ultrasonography for brain tumors: A pictorial essay on the technique, its utility, and its benefits in neuro-oncology. *Ultrasonography*. Published online 2020. doi:10.14366/usg.20044

58. Yu SQ, Wang JS, Chen SY, et al. Diagnostic Significance of Intraoperative Ultrasound Contrast in Evaluating the Resection Degree of Brain Glioma By Transmission Electron Microscopic Examination. *Chinese Medical Journal*. 2015;128(2). doi:10.4103/0366-6999.149194
